# Announcement: The Third International Symposium on Speciation of Elements in Biological, Environmental and Toxicological Sciences

**DOI:** 10.1155/MBD.1996.163

**Published:** 1996


### The Third International Symposium on SPECIATION OF ELEMENTS IN BIOLOGICAL, ENVIRONMENTAL AND TOXICOLOGICAL SCIENCES

### The Torresian Resort Port Douglas Queensland, Australia, September 15-19, 1997

### THE SYMPOSIUM IS ORGANIZED BY:

The University of New South Wales (Sydney, Australia)
The National Institute of Occupational Health (Oslo, Norway)
The Institute of Environment and Health
(Universities of Toronto and McMaster, Canada)
MAFF CSL Food Science Laboratory (Norwich, UK)

### INVITATION AND CALL FOR PAPERS

The Organizing Committees extend an invitation to all individuals involved in element research or its applications. A major goal of the Symposium is to facilitate interdisciplinary and intersector discussion about all aspects of elements requiring an understanding of speciation, including: analytical chemistry, geochemistry, biochemistry, environmental sciences, essentiality and nutrition, medical uses, occupational hygiene, human toxicology and regulatory aspects. A small number of travel scholarships will be provided to encourage overseas graduate students to attend and participate.

### THE SCIENTIFIC PROGRAMME

The Symposium programme will comprise four days of oral presentations, posters and discussion. All presenters will be asked to focus on new developments in research. Oral presentations (invited or submitted) will be thirty or twenty minutes in duration.
The tradition established by the First and Second Symposia in this series (both held in Loen, Norway in June 1991 and 1994, respectively) of assigning a prominent and central role to poster presentations is to continue. Consequently, concurrent sessions organized by themes will be held after the formal viewing period to encourage in depth feedback and discussion. In these special discussion groups, each author will be allotted 5 minutes for an oral presentation of the salient features of her or his poster.

### CONFERENCE PROCEEDINGS

As for the previous Speciation Symposia (see The Analyst **117**: 549-691;**120**: 29-30N, 583-763), all papers presented as posters or lectures may be submitted as full papers for publication in a special issue of The Analyst, subject to the normal review procedures of this journal.

### SYMPOSIUM LOCATION AND DETAILS

The venue of the Symposium is The Torresian Resort Port Douglas, Australia. This tropical Queensland location is situated near Cairns between the Great Barrier Reef and the Daintree rainforest. A Symposium Package rate has been arranged: AUD $155 (per person, per night, twin share) and AUD $225 (single occupancy), and includes accommodation (Garden View Room), all meals, and morning and afternoon teas. A limited amount of less expensive accommodation (room and board) will be available. The Symposium schedule is as follows: reception, Monday evening, Sept. 15; Tuesday to Friday inclusive, scientific sessions 8:00-12:30 and 13:30-15:30. This scheduling allows the participants to join the XXX Colloquium Spectroscopium Internationale (CSI) in Melbourne, held Sunday Sept. 21 to Friday, Sept. 26, 1997.

### SOCIAL PROGRAMME

All participants and accompanying persons are invited to the Symposium Reception on Monday evening, Sept. 15 and the Dinner on Friday evening, Sept. 19. Because of the numerous attractions available (e.g., swimming, all other water sports, cruises, canoeing, hiking, horse riding, etc.), no other formal social events are planned. However, please note that for each full day of scientific sessions, the period 15:30 onwards will be set aside for the enjoyment of the mentioned activities by all. Port Douglas has a comfortable, year round, tropical climate. Day tours to the Outer Barrier Reef are available.

### REGISTRATION FEE

The registration fee per delegate is AUD $480 and includes the Symposium Dinner cost. The registration fee for students is AUD $150.

### Preliminary Registration Form

Third Speciation Symposium, September 15-19, 1997
The Torresian Resort Port Douglas, Australia
O I am interested in participating
O Please put me on your mailing list
I expect to be accompanied by
O Partner . . . . . . . . . . . . . . . . . Number of children
O I intend to offer an oral presentation
O I intend to offer a poster presentation
Provisional title of poster/paper: . . . . . . . . . . . . . . . . . . . . . . . . . . . . . . . . . . . .
. . . . . . . . . . . . . . . . . . . . . . . . . . . . . . . . . . . . . . . . . . . . . . . . . . . . . .
Dr. / Mr. / Mrs. / Miss . . . . . . . . . . . . . . . . . . . . . . . . . . . . . . . . . . . . . . . . .
University/Institute/Company:. . . . . . . . . . . . . . . . . . . . . . . . . . . . . . . . . . . . . .
. . . . . . . . . . . . . . . . . . . . . . . . . . . . . . . . . . . . . . . . . . . . . . . . . . . . . .
Address:. . . . . . . . . . . . . . . . . . . . . . . . . . . . . . . . . . . . . . . . . . . . . . . . . .
. . . . . . . . . . . . . . . . . . . . . . . . . . . . . . . . . . . . . . . . . . . . . . . . . . . . . .
Telephone:. . . . . . . . . . . . . . . . . . . . . . . . . . . . . . . . . . . . . . . . . . . . . . . .
Fax:. . . . . . . . . . . . . . . . . . . . . . . . . . . . . . . . . . . . . . . . . . . . . . . . . . . .
E-mail: . . . . . . . . . . . . . . . . . . . . . . . . . . . . . . . . . . . . . . . . . . . . . . . . . .

Please return this registration as soon as possible to the Local Secretariat (See below)

### ORGANIZING COMMITTEES

### Programme Committee

* **Helen M. Crews**, MAFF CSL Food Science Laboratory, Norwich, U.K.
* **Jarda P. Matousek**, The University of New South Wales, Sydney, Australia.
* **Evert Nieboer** (Programme Co-Chair), McMaster University, Hamilton, Canada.
* **Yngvar Thomassen** (Programme Co-Chair), National Institute of Occupational Health, Oslo, Norway.

### Local Committee

* **Graeme E. Batley**, CSIRO Centre for Advanced Analytical Chemistry, Lucas-Heights
* **R. (Dick) J. Finlayson**, The University of New South Wales, Sydney
* **D. Brynn Hibbert**, The University of New South Wales, Sydney
* **Jarda P. Matousek** (Conference Chair), The University of New South Wales, Sydney

### SECRETARIATS

### Local (Registration)

Third Speciation Symposium, c/o Dr. **J.P. Matousek**, Department of Analytical Chemistry, The University of New South Wales, Sydney NSW 2052, Australia
Tel: 61-2-3854713 61-2-4512322 (home) Fax: 61-2-3856141
E-mail: J.Matousek@unsw.edu.au

### Programme

Third Speciation Symposium, c/o Dr. **E. Nieboer,** McMaster University, Department of Biochemistry, 1200 Main Street West, Hamilton, ON Canada, L8N 3Z5

Tel: 905-525-9140, x22048 Fax: 905-522-9033
E-mail: Nieboere@fhs.csu.McMaster.ca

